# Endemic Angiostrongyliasis, Rio de Janeiro, Brazil

**DOI:** 10.3201/eid1707.101822

**Published:** 2011-07

**Authors:** Raquel O. Simões, Fernando A. Monteiro, Elizabeth Sánchez, Silvana C. Thiengo, Juberlan S. Garcia, Sócrates F. Costa-Neto, José L. Luque, Arnaldo Maldonado

**Affiliations:** Author affiliations: Fundação Oswaldo Cruz, Rio de Janeiro, Brazil (R.O. Simões, F.A. Monteiro, E. Sánchez, S.C. Thiengo, J.S. Garcia, S.F. Costa-Neto, A. Maldonado Jr.);; Universidade Federal Rural do Rio de Janeiro, Seropédica, Brazil (R.O. Simões, J.L. Luque)

**Keywords:** parasites, rats, rat lung worm, Rio de Janeiro, Angiostrongylus cantonensis, Rattus norvegicus, letter

**To the Editor:** The nematode *Angiostrongylus cantonensis* (rat lung worm), a zoonotic parasite that can accidentally infect humans and cause eosinophilic meningoencephalitis, has the Norway rat (*Rattus norvegicus*) as one of its most frequent definitive vertebrate hosts (*1*). Adult worms live in the pulmonary arteries of the definitive hosts, which excrete first-stage larvae in their feces. Intermediate hosts, such as snails and slugs, are infected by first-stage larvae, which reach the infective third stage after 2 molts. Third-stage larvae are then ingested by rats as they feed on the intermediate hosts, thus closing the life cycle. Humans become infected by eating raw or undercooked snails and slugs and through paratemic hosts and vegetables contaminated with infected snail mucus (*2*).

In Brazil, the first 3 documented cases of eosinophilic meningoencephalitis occurred in 2007 in 2 cities in the southeastern state of Espírito Santo (*3*). In 2009, a new case was reported in Pernambuco in the northeast region (*4*). Only intermediate hosts have been found naturally infected with rat lung worm in Brazil. Infected terrestrial and freshwater snails of the species *Achatina fulica*, *Sarasinula marginata*, *Subulina octona*, and *Bradybaena similaris* in Espírito Santo; *A. fulica* and *Pomacea lineata* in Pernambuco; and *A. fulica* in Rio de Janeiro and Santa Catarina have been reported (*3**,**5**,**6*). Thus, because of the recent cases of eosinophilic meningoencephalitis in Brazil and the occurrence of naturally infected *A. fulica* snails in Rio de Janeiro, we investigated the existence of potential natural reservoirs for the parasite in São Gonçalo.

São Gonçalo (22°48′26.7′′S, 43°00′49.1′′W) is a densely populated city (≈1 million inhabitants) with a tropical Atlantic climate (14°C–35°C) that is part of the metropolitan region of Rio de Janeiro. Two collections were made in March and June 2010. Forty live traps (20 Tomahawk [Tomahawk Live Trap Company, Tomahawk, WI, USA] and 20 Sherman [H.B. Sherman Traps Inc., Tallahassee, FL, USA] traps) were placed along two 30-m transects for 4 consecutive nights (Brazilian Institute of Environment and Renewable Natural Resources license no. 2227–1/2010) in an urban area where *A. fulica* snails had been collected in high numbers. Twenty-seven Norway rats (16 males) were captured. We collected 265 adult lung worms from the pulmonary arteries of the captured animals, fixed the worms in 70% ethanol, and taxonomically identified them as *A. cantonensis* on the basis of the large size of the spicules and the patterns of the bursal rays (*7*). Voucher specimens have been deposited in the Helminthological Collection of the Oswaldo Cruz Institute (no. 35712). Nineteen (74%) rats were infected; mean intensity and mean abundance were 13.52 ± 2.36 and 9.81 ± 1.96, respectively.

To confirm the morphologic identification of the *Angiostrongylus* specimens obtained, a DNA bar coding approach was used. DNA was extracted from 3 ethanol-preserved adult worms previously recovered from the pulmonary arteries of a naturally infected Norway rat, PCR-amplified, sequenced for a partial region of the COI gene (*8*), and subsequently compared with available GenBank *Angiostrongylus* spp. sequences. The three 360-bp COI sequences obtained (GenBank accession no. HQ440217) were Clustal-aligned (www.clustal.org) with homologous COI fragments of *A. cantonensis* (GenBank accession no. GQ398121), *A. vasorum* (GenBank accession nos. EU493162, EU493163, EU493166, EU493167), and *A. costaricensis* (GenBank accession no. GQ398122) and subjected to phylogenetic analysis. *Ancylostoma tubaeforme* (GenBank accession no. AJ407940) was used as the outgroup. Haplotypes for *A. vasorum* isolates from Brazil (*A. vasorum* 5421, 5641, and 5642) were reconstructed from published information (*9*) and included in the alignment. We used MEGA4 (www.megasoftware.net) to construct a neighbor-joining phylogenetic tree based on Kimura 2-parameter (K2-p) distances (Figure). The 3 *A. cantonensis* specimens from São Gonçalo, Rio de Janeiro, yielded a single haplotype, which formed a clade with the *A. cantonensis* haplotype from the People’s Republic of China with low genetic distance (K2-p 0.038) and high bootstrap support (98), thus confirming the morphologic identification. Comparisons with the other 2 *Angiostrongylus* species yielded higher genetic distance values (K2-p 0.120, with *A. vasorum*, and 0.149, with *A. costaricensis*).

**Figure:**
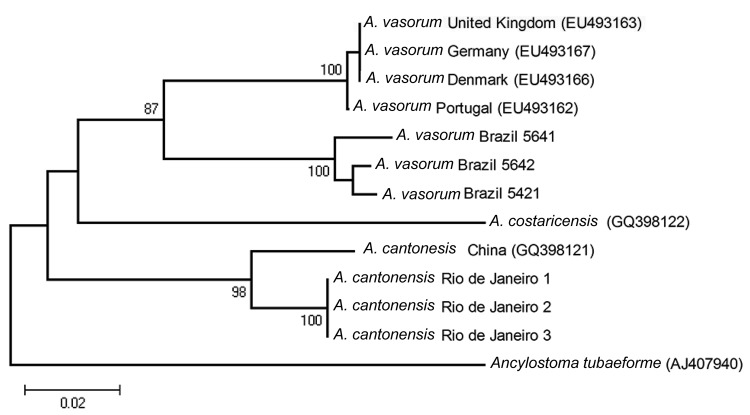
Neighbor-joining phylogenetic tree based on Kimura 2-parameter (K2-p) distances that includes all *Angiostrongylus* COI sequences in GenBank and the sequences obtained from 3 *Angiostrongylus* specimens recovered from the pulmonary arteries of a naturally infected Norway rat (*Rattus norvegicus*) from São Gonçalo, Rio de Janeiro, Brazil, 2010. The specimens yielded 1 haplotype, which clustered together with the *A. cantonensis* haplotype from the People’s Republic of China with a low genetic distance (K2-p 0.038). Scale bar indicates 0.02 K2-p genetic distance.

These results indicate that *A. cantonensis* lung worm infection is enzootic among the exotic Norway rat population in the region studied. The natural infection rate of 74% is the second highest reported among 14 severely *A. cantonensis* infection–endemic regions (*2*). These findings, together with the observation of dense populations of *A. fulica* snails in urban areas of the country (*10*), call attention to the risk for disease transmission to humans, given that Norway rats also are likely to be present in these areas.

Local residents should be informed about disease transmission and prevention, and physicians should consider *A. cantonensis* lung worm infection in the differential diagnosis when appropriate. Although public health authorities should consider implementation of surveillance and control strategies to reduce the populations of snail and rat hosts, a better understanding is needed of the epidemiologic significance of these findings, which can be attained through studies to identify human cases of eosinophilic meningitis in the region.
